# Social isolation and anxiety disorders during COVID-19: a systematic review

**DOI:** 10.3389/fpubh.2025.1688239

**Published:** 2025-12-05

**Authors:** Fetoon Massad Saud Al-Otaibi, Motaz Thaieb Alotaibi, Nadia Altamimi, Sadeq Abu-Dawas, Ahmed Yaqinuddin, Khaled Alkattan

**Affiliations:** 1 College of Medicine, Alfaisal University, Riyadh, Saudi Arabia; 2 Department of Psychology, College of Social Sciences, Imam Mohammad Ibn Saud Islamic University, Riyadh, Saudi Arabia; 3 Department of Clinical Psychology, King Fahad Medical City, Riyadh, Saudi Arabia

**Keywords:** COVID-19, social isolation, anxiety disorders, mental health, pandemic, social distancing, systematic review

## Abstract

**Background:**

This systematic review examines the relationship between prolonged social isolation during the COVID-19 pandemic and anxiety levels in adults, with specific focus on social anxiety. It highlights that enforced distancing measures like lockdowns and reduced social contact significantly contributed to a global rise in psychological distress and anxiety disorders.

**Objective:**

To synthesize recent evidence on how social isolation influenced anxiety levels in the general adult population during the COVID-19 pandemic.

**Methodology:**

This study followed a qualitative systematic review design. Relevant literature was identified through searches in databases including PubMed, MEDLINE, Web of Science, SCOPUS, and others, using combinations of MeSH terms and keywords. Data extraction and quality assessment followed the PRISMA guidelines and used the Downs and Black Checklist to evaluate methodological quality.

**Results:**

Seven studies were included, with a total of 3,014 participants. Findings revealed a consistent positive association between social isolation and anxiety. Perceived isolation was a stronger predictor of anxiety than objective isolation. Older adults showed higher vulnerability when isolated or lacking social support. Students and young adults also experienced elevated anxiety, especially when living alone or facing COVID-related stressors.

**Conclusion:**

Social isolation during the pandemic significantly contributed to increased anxiety symptoms across global adult populations. Public health efforts should target loneliness and promote sustainable social connectedness to mitigate long-term psychological consequences.

## 1 Introduction

The COVID-19 pandemic caused significant disruptions to daily life worldwide, with profound negative effects on global mental health (1, 2). A key consequence has been a substantial increase in anxiety disorders, with global cases rising by an estimated 76.2 million—a 25.6% increase (3). A return to pre-pandemic mental health levels appears unlikely in the near future (4), highlighting the need to understand the pandemic's lasting psychological impacts.

Government-mandated public health measures, such as quarantines, lockdowns, the closure of workplaces and schools, and physical distancing, drastically reduced face-to-face social interactions (5, 6). For the general population, this enforced isolation represented a sudden and prolonged shift in social behavior. Given that social connection is a fundamental human need (7), the abrupt severance of these ties posed a significant threat to psychological wellbeing. A growing body of evidence has documented the detrimental psychological effects of these measures, including increased levels of stress, depression, and anxiety across diverse populations (8, 9).

While these effects were widespread, the impact on anxiety symptoms is of particular interest. Anxiety often arises in response to perceived threats and uncertainty, both of which were hallmarks of the pandemic (10). The specific role of social isolation, as distinct from other pandemic-related stressors like health fears or financial insecurity, requires focused examination. Preliminary research suggests that the lack of social support and feelings of loneliness during lockdowns were key drivers of worsening anxiety (11).

Therefore, this systematic review aims to synthesize recent evidence from 2022 to 2025 to specifically investigate the relationship between social isolation and anxiety disorders in the general adult population during the COVID-19 pandemic. By focusing on this relationship, the review seeks to clarify the extent to which isolation itself contributed to the observed surge in anxiety, informing future public health strategies for mental health promotion during crises.

## 2 Methodology

### 2.1 Study design

This systematic review adopted a narrative synthesis approach to examine original research articles that explored the relationship between social isolation during the COVID-19 pandemic and anxiety disorders among individuals aged 18 and above. The aim was to synthesize findings that shed light on how enforced social distancing measures influenced anxiety disorders in the general adult population.

### 2.2 Search strategy

A comprehensive literature search was conducted across several electronic databases, including PubMed, MEDLINE, SCOPUS, and Web of Science. The search strategy utilized a combination of Medical Subject Headings (MeSH) and keywords to maximize retrieval. The specific search terms included: “anxiety,” “social isolation,” “COVID-19,” “pandemic,” “mental health,” “social distancing,” and “coronavirus.” These terms were combined using the Boolean operators ‘AND' and ‘OR' to refine the search. The search was limited to studies published between January 2022 and March 2025 to capture the most recent evidence produced after the initial acute phase of the pandemic. To ensure a comprehensive search, the reference lists of all included studies were manually screened for additional relevant publications.

### 2.3 Eligibility criteria

#### 2.3.1 Inclusion criteria

Studies were included if they were: (12) original research articles published in English; (13) involved participants aged 18 years or older from the general population; (7) explicitly investigated the association between social isolation and anxiety during the COVID-19 pandemic, as indicated in the title or abstract; and (5) used a validated scale or instrument to measure anxiety symptoms (e.g., GAD-7, DASS-21).

#### 2.3.2 Exclusion criteria

Studies were excluded if they: (12) were not original research (e.g., reviews, editorials, conference abstracts); (13) focused on specific high-risk subgroups (e.g., healthcare workers, adolescents, pregnant women, or individuals with pre-existing chronic physical or mental illnesses); (7) lacked quantifiable data on anxiety outcomes; (5) were duplicates; or (14) relied solely on grey literature. The exclusion of specific populations was implemented to minimize confounding variables and maintain a focus on the general adult population.

### 2.4 Study selection

The study selection process was conducted in accordance with the PRISMA guidelines (15). Initially, all identified records were screened by title and abstract by two independent reviewers to assess their relevance based on the eligibility criteria. The full texts of potentially eligible studies were then retrieved and reviewed independently by the same two reviewers. Any disagreements regarding inclusion were resolved through discussion until a consensus was reached.

### 2.5 Data extraction

Data from the included studies were extracted using a standardized data extraction form. The extracted information included: author(s), year of publication, country of study, study design, sample size, participant characteristics (e.g., mean age, gender distribution), social isolation measurement, anxiety measurement tool, and key findings related to the isolation-anxiety relationship.

### 2.6 Quality assessment

The methodological quality of the included observational studies was assessed using the adapted Downs and Black Checklist. This tool evaluated key aspects of study quality, including the clarity of the research aim, description of the participant population, measurement of main outcomes, and control for confounding. Each study was rated across specific criteria, with a score of “1” indicating the criterion was met, “05” partially met, and “0” not met.

## 3 Results

**Figure 1:**
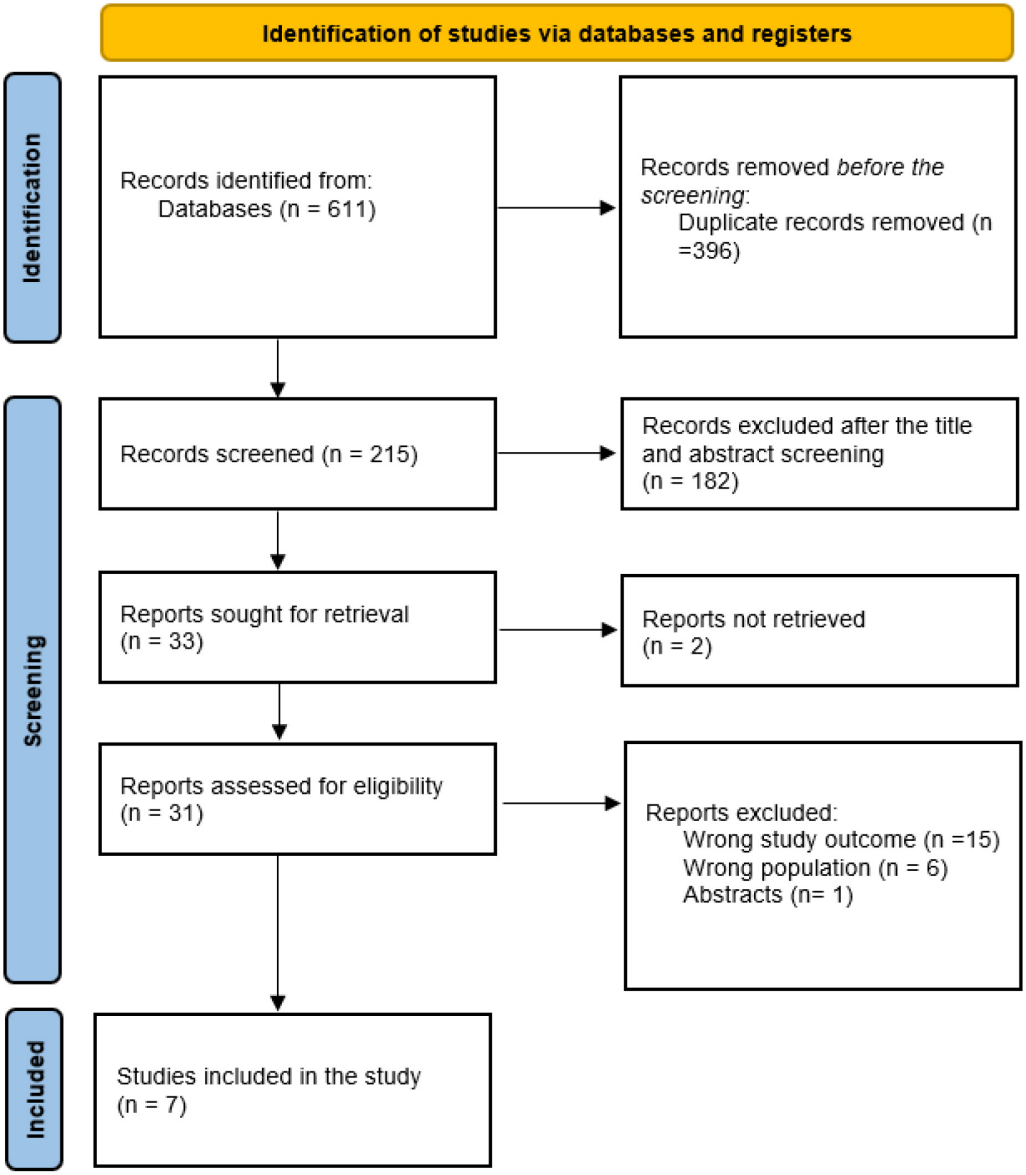
Search summary illustrated in PRISMA flowchart.

The systematic search and selection process, detailed in the PRISMA flowchart (Figure 1), initially identified 611 records. After the removal of 396 duplicates, 215 articles underwent title and abstract screening. A total of 182 records were excluded for not meeting the eligibility criteria, leaving 31 full-text articles to be assessed for eligibility. Upon detailed review, seven studies fulfilled all inclusion criteria and were included in the final evidence synthesis and analysis.

### 3.1 Characteristics of included studies

The seven included studies comprised a total of 3,014 participants. All studies employed a cross-sectional design. The studies were conducted across diverse geographical locations, including Australia, Brazil, India, Iran, Italy, and Spain, providing a broad international perspective. A summary of the study characteristics, including sociodemographic details, measures of social isolation and anxiety, and main outcomes, is presented in Table 1.

**Table 1:** Summary of included studies: sociodemographic, isolation, and anxiety measures.

**Study ID**	**Country**	**Study design**	**Sociodemographic**	**Social isolation scale**	**Anxiety scale**	**Main outcomes**
Mann et al. (17)	Australia	Cross-sectional	Cases: 578 mean age: 39.2 females: 344 (59.5%)	Self-reported	DASS-21^*^	During the COVID era, social isolation was linked with increased psychological distress, particularly anxiety. While objective isolation showed a weak negative relationship with anxiety, perceived isolation was more strongly and positively associated with anxiety symptoms.
Mahdie et al. (20)	Iran	Cross-sectional	Cases: 300 mean age: 71 females: 177 (59%)	LSNS	CDAS	Social isolation is a significant predictor of COVID-19-related anxiety among older adults, alongside factors like marital status and experiencing COVID-19-related deaths within the family.
Sousa et al. (21)	Brazil	Cross-sectional	Cases: 450 mean age: 67.2 females: N/R	Self-reported	GAI	Social isolation during the COVID-19 pandemic contributed to the development of anxiety and depressive disorders among older adult Brazilians. Key factors included low educational levels and being divorced.
Verma and Mehta (19)	India	Cross-sectional	Cases: 438 mean age: 22.2 females: 307 (70.1%)	Self-reported	GAD-7^*^	A large proportion of young adults experienced varying levels of anxiety during the COVID-19 pandemic, with social isolation emerging as a key contributing factor. Living alone and knowing someone infected with the virus further increased anxiety levels.
Jantara et al. (16)	Brazil	Cross-sectional	Cases: 147 mean age: 23.4 females: 137 (93.2%)	Self-reported	DASS-21^*^	Feeling socially accepted was associated with better mental health, while loneliness was strongly linked to higher anxiety, stress, and depression.
da Cruz et al. (14)	Spain	Cross-sectional	Cases: 76 mean age: 18.3 females: 0	Self-reported	SCAT	Confinement during the COVID-19 pandemic led to increased anxiety levels among elite soccer athletes, even though their sleep quality and daytime sleepiness remained stable.
Mojsa-Kaja et al. (18)	Italy	Cross-sectional	Cases: 1025 mean age: 24 females: N/R	Self-reported	STAI-S	A clear link was found between social isolation and increased levels of anxiety, depression, and insomnia during the COVID-19 pandemic. Insomnia was closely connected to both anxiety and depression.

N/R = Not Reported; ^*^Full names of acronyms: DASS-21 = Depression, Anxiety and Stress Scales - 21 Items; LSNS = Lubben Social Network Scale; CDAS = Corona Disease Anxiety Scale; GAI = Geriatric Anxiety Inventory; GAD-7 = Generalized Anxiety Disorder 7-item scale; SCAT = Sport Competition Anxiety Test; STAI-S = State-Trait Anxiety Inventory - State subscale.^*^

### 3.2 Synthesis of findings

Across all reviewed studies, a consistent positive association was identified between social isolation and increased anxiety levels during the COVID-19 pandemic. Individuals who perceived themselves as socially disconnected or lonely reported significantly higher levels of psychological distress, particularly anxiety symptoms (16–19). This relationship was evident across various populations, including young adults, university students, older adult individuals, and elite athletes.

A key finding that emerged from the synthesis was the distinction between objective and perceived isolation. Perceived loneliness was consistently identified as a stronger predictor of anxiety symptoms than objective measures of physical isolation alone (17). The impact of isolation was especially pronounced in groups with limited social support or disrupted routines, indicating that social connection acted as a buffer against anxiety.

The review also identified specific vulnerable subgroups within the general population. Older adults (e.g., mean age 67–71) were found to experience higher levels of anxiety and depression when they were isolated, divorced, or had low educational levels (20, 21). Conversely, younger adults and students also reported elevated anxiety, particularly when living alone or knowing someone infected with COVID-19 (19). The feeling of being socially accepted was linked to better mental health outcomes, while loneliness was strongly associated with higher levels of stress, anxiety, and depression (16).

Furthermore, the interconnectedness of mental health symptoms was noted. While one study involving elite athletes reported stable sleep quality despite increased anxiety (14), another clearly linked insomnia with both anxiety and depression, suggesting a complex, intertwined relationship among these conditions during periods of isolation (18).

### 3.3 Risk of bias assessment

**Figure 2:**
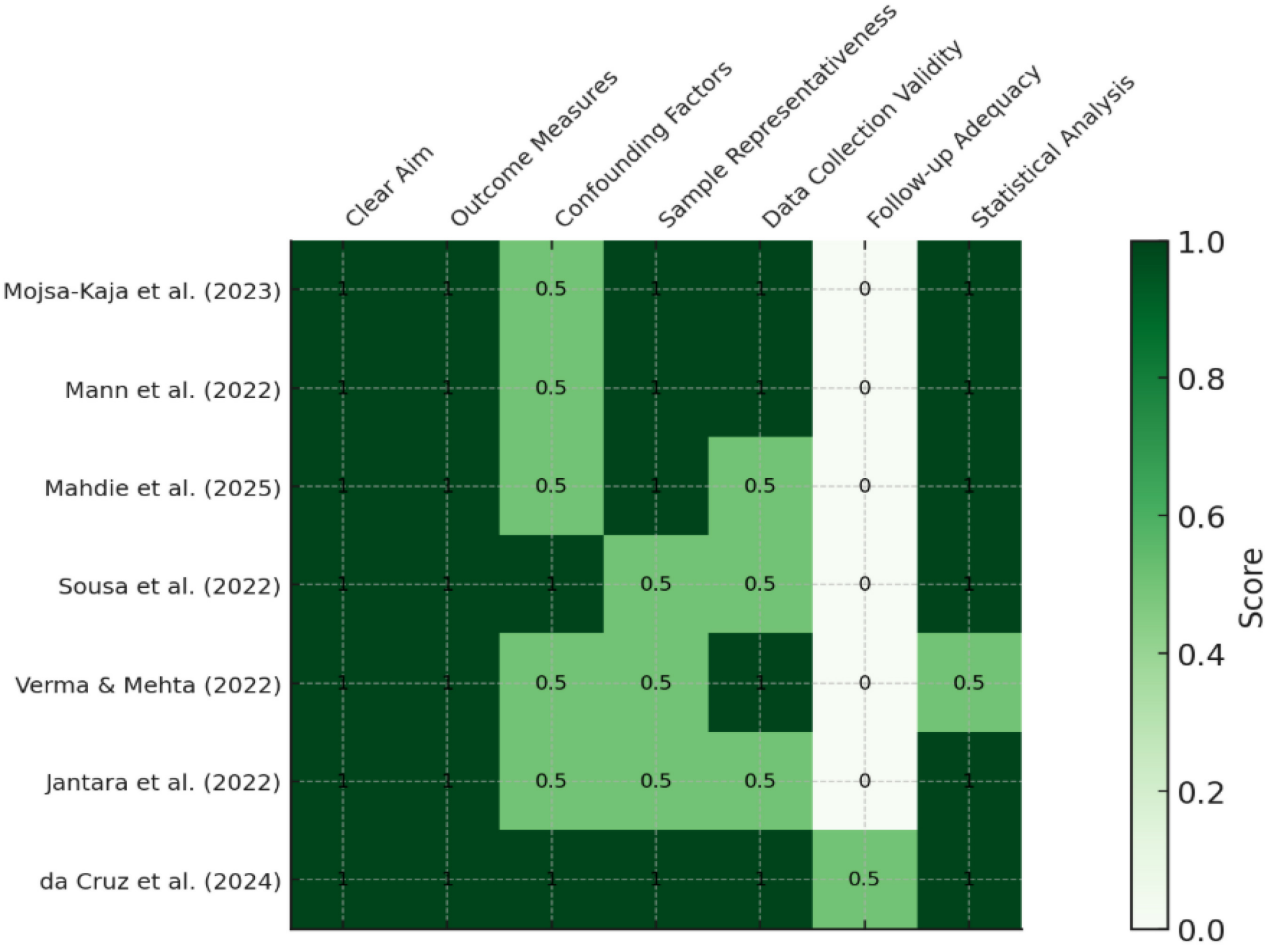
Risk of bias assessment.

The methodological quality of the included studies, as assessed by the adapted Downs and Black checklist, is presented in Figure 2. The shading reflects the strength of methodological quality across seven core criteria.

## 4 Discussion

The present synthesis of seven cross-sectional studies confirms a consistent association between social isolation and elevated anxiety levels during the COVID-19 pandemic across diverse populations, including young adults, the older adult, college students, and elite athletes. A central finding of this review is that perceived or subjective isolation was more strongly linked to psychological distress than objective measures of social disconnection. This distinction, which was explicitly measured in the included studies using self-report scales for loneliness vs. objective counts of social contacts, underscores that loneliness is a more critical determinant of mental health outcomes than the mere absence of social contact alone (17). This aligns with a broader systematic review by Dos Santos and team, which emphasized that recognizing the extent and impact of the pandemic on individuals' lives, including sociodemographic factors, is essential to understanding its psychological toll (22).

In several of the included studies, perceived social isolation significantly predicted anxiety, with mediating variables influencing the severity of outcomes. For instance, the study by Mojsa-Kaja and team highlighted the mediating role of insomnia, which served as a bridge between isolation and psychological distress among young adults (18). Similarly, studies involving older adult populations highlighted social isolation as a predictor of anxiety and depressive symptoms, especially when compounded by low educational attainment and marital separation (20, 21). These findings are consistent with other reviews; Akinlotan and Jalo reported that extended loneliness negatively impacted the mental health of older adults, intensifying anxiety and fear (13), while Su and team found that the prevalence of loneliness and social isolation among older adults increased as the pandemic persisted (23).

While our review focused on the general adult population, the findings naturally highlight variations in vulnerability across different subgroups within that population. The data extracted from the included studies indicate that older adults and young students were particularly affected. For older adults, mitigating loneliness often required maintaining remote connections. However, as noted in other literature, traditional communication tools like phones often lacked the interpersonal engagement of digital platforms (24), and older adults in low- and middle-income countries faced significant barriers due to limited digital literacy and financial means (25–27). This highlights how the pandemic exacerbated existing social and digital inequalities, directly impacting mental health.

Similarly, research among university students identified social isolation as a central stressor linked to anxiety and depression (16, 19). The effects appeared more severe among students living alone or with reduced access to social support. This is supported by Martins and team, who recommended that post-pandemic interventions for students focus on promoting healthy routines and that universities establish proactive support mechanisms to foster a secure and inclusive atmosphere (28).

The findings of this review underscore the urgent need for public health interventions that address both the objective and subjective dimensions of social isolation. Clinicians and mental health professionals should be especially attentive to perceived loneliness, which emerged as a stronger predictor of anxiety than physical isolation. For older adults, interventions may need to prioritize digital literacy programs and accessible communication platforms to enhance social connectedness. For younger adults and students, structured routines, peer support systems, and accessible mental health services could mitigate anxiety associated with isolation. As the mental health impact of the pandemic may persist beyond its physical containment, targeted psychological support should remain a long-term priority.

## 5 Strengths and limitations

This systematic review is strengthened by its clear focus, rigorous methodology, and inclusion of only recent studies (2022–2025), ensuring relevance to ongoing post-pandemic contexts. By excluding populations with pre-existing medical or psychiatric conditions, the review isolates the psychological impact of social isolation in the general adult population, reducing confounding variables. The use of standardized tools (e.g., DASS-21, GAD-7) and quality assessment via the Downs and Black checklist further enhances methodological reliability. Moreover, the inclusion of diverse global populations (e.g., Brazil, Iran, India, Australia) offers broad insight into how cultural and demographic factors shape the experience of anxiety during isolation.

Despite these strengths, several limitations should be acknowledged. First, all included studies employed a cross-sectional design, limiting causal inference regarding the relationship between social isolation and anxiety. The reliance on self-reported measures also introduces potential reporting bias, especially in cultures where mental health stigma may affect participant disclosure. Furthermore, while the review excluded studies with medically vulnerable populations, the included samples still varied in terms of age, education, and socioeconomic background, which may limit the generalizability of findings to more homogenous subgroups. Lastly, the lack of longitudinal data restricts understanding of how anxiety symptoms evolved over different phases of the pandemic.

## 6 Conclusion

In conclusion, this systematic review confirms a robust and consistent association between social isolation—particularly perceived isolation—and anxiety disorders among adults during the COVID-19 pandemic. Vulnerable groups such as the older adult, students, and individuals with low social support were particularly affected. Although social distancing was necessary for infection control, it came at a significant psychological cost. Addressing the mental health aftermath of the pandemic requires sustained, context-sensitive efforts to rebuild social connections and provide accessible mental health support, especially for those who experienced prolonged isolation.

## Data Availability

The original contributions presented in the study are included in the article/supplementary material, further inquiries can be directed to the corresponding author.
